# The application and limitations of exposure multiplication factors in sublethal effect modelling

**DOI:** 10.1038/s41598-022-09907-1

**Published:** 2022-04-11

**Authors:** Neil Sherborne, Tjalling Jager, Benoit Goussen, Marie Trijau, Roman Ashauer

**Affiliations:** 1 Syngenta, Jealott’s Hill International Research Centre, Bracknell, Berkshire, RG42 6EY UK; 2 DEBtox Research, Stevensweert, The Netherlands; 3 Ibacon GmbH, Roßdorf, Germany; 4 Syngenta Crop Protection AG, 4058 Basel, Switzerland; 5 Department of Environment and Geography, University of York, Wentworth Way, Heslington, York, YO10 5NG UK

**Keywords:** Ecological modelling, Ecological modelling, Agroecology

## Abstract

Thanks to growing interest and research in the field, toxicokinetic–toxicodynamic (TKTD) models are close to realising their potential in environmental risk assessment (ERA) of chemicals such as plant protection products. A fundamental application is to find a multiplicative scale factor which—when applied to an exposure profile—results in some specified effect relative to a control. The approach is similar to applying assessment factors to experimental results, common in regulatory frameworks. It also relies on the same core assumption: that increasing the scaling always produces more extreme effects. Unlike experimental approaches, TKTD models offer an opportunity to interrogate this assumption in a mathematically rigorous manner. For four well-known TKTD models we seek to prove that the approach guarantees a unique scale factor for any percentage effect. Somewhat surprisingly, certain model configurations may have multiple scale factors which result in the same percentage effect. These cases require a more cautious regulatory approach and generate open biological and mathematical questions. We provide examples of the violations and suggest how to deal with them. Mathematical proofs provide the strongest possible backing for TKTD modelling approaches in ERA, since the applicability of the models can be determined exactly.

## Introduction

Assessing the ecological consequences of introducing chemical compounds, such as plant protection products (PPPs), into an environment requires some form of environmental risk assessment (ERA). At the individual level, an assessment compares the effects that exposure to the substance has on the organism to the level of exposure that it is likely to experience in the field. Traditionally, laboratory studies on standard species under relatively constant exposure have been used to derive certain summary statistics^1^. For instance, a no observed effect concentration (NOEC) or an estimated concentration where $x\%$ effects relative to the control performance occur ($\hbox {EC}_{x}$). These statistical results are then compared to a predicted peak or time-weighted exposure concentration to derive a toxicity exposure ratio (TER). The final step is to compare the TER to some assessment factor (AF). The AF is designed to cover uncertainties relating to extrapolation to different species, conditions and exposure. If the TER is greater than the AF then the use case of the PPP is considered low risk. Setting aside the known methodological flaws in NOECs and $\hbox {EC}_{x}$ values^2^, the approach cannot account for exposures which fluctuate over time. Variable exposure is typical in the field and can significantly alter the effects on the individual. Data collection and modelling methods exist which generate time series of concentrations present in the environment over time. For instance, in the aquatic environment, surface water models predict concentration levels over time in water bodies near treated fields. We refer to these time series concentrations as exposure profiles, since they determine the variable level of concentration organisms will be exposed to over time. ERA methods which accurately incorporate time-variable exposure are therefore valuable. However, it is impossible to test in the laboratory every exposure profile which may occur in the field.

In recent years, there has been growing interest in mechanistic models which can explain the effects of exposure to a harmful substance on an individual, population or community of organisms^3–7^. At the individual level, toxicokinetic–toxicodynamic (TKTD) models describe how given exposure conditions ultimately translate to effects on the organism over time^8^. Due to their mechanistic structure it is possible to extrapolate to predict effects under a different set of conditions, in particular to untested, time-variable exposure profiles. This ability to take into account the whole exposure profile is a great strength of TKTD models.

Within ERA, the pioneer of these models is the General Unified Threshold model of Survival (GUTS)^4^, a TKTD model for lethal effects which has been approved by the European Food Safety Authority (EFSA) for use in ERA of aquatic organisms^7^. The GUTS model can be calibrated on standard toxicity tests and used to predict the survival probability of an organism exposed to any exposure profile via the exposure multiplication factor (EMF) method. Ashauer et al.^9^ first introduced the concept of the EMF in ERA, although they referred to it as the multiplicative margin of safety. An EMF is a scale-factor applied to the exposure profile. Through iterative methods, it is possible to find an EMF value which corresponds exactly to some chosen level of effects. The approved procedure for GUTS in ERA uses this approach to identify the lethal profile for $x\%$ effects ($\hbox {LP}_{x}$)^7^. The $\hbox {LP}_{x}$ has a similar interpretation as the TER, since it tells us the extent to which the threshold for $x\%$ toxicant induced mortality is greater than the predicted exposure. However, now that extent is based on the entire exposure profile, not a single value. Therefore, risk assessment decisions can compare an $\hbox {LP}_{x}$ to the same AF values used with the TER. Although other measures for the potential effects of chemical exposure based on TKTD models exist^5^, the presumption is that the EMF method will be transferred to other TKTD models^7^. For models of sublethal effects, the term effective profile ($\hbox {EP}_{x}$) is equivalent to the $\hbox {LP}_{x}$.

Despite the EMF method gaining acceptance, little attention has been paid to the core assumption that the method makes. Namely, that scaling (multiplying) an exposure profile will always identify a unique $\hbox {LP}_{x}$ (or $\hbox {EP}_{x}$) for any valid *x* (0–100%). While this may seem intuitively true it is nonetheless an assumption, especially for more complex models and variable exposure profiles which may offer significant potential for organisms to repair damage and recover from sublethal effects. However, it is important to note that similar assumptions are made implicitly for all real-world experiments, and have also not been thoroughly scrutinised. It is thanks to modelling that we can test these assumptions at all. Unlike real-world experiments, TKTD models allow us to interrogate arbitrary exposures with full knowledge of all state variables at all times. This knowledge means that general rules can be identified and proved. A mathematical proof of existence and uniqueness of the $\hbox {LP}_{x}$ (or $\hbox {EP}_{x}$) value for the TKTD model would put the method on the most solid ground possible.

Recently, Baudrot and Charles^10^ calculated expressions for the $\hbox {LP}_{x}$ values for the reduced forms of the GUTS model. Their results implicitly show that the $\hbox {LP}_{x}$ exists and is unique for any non-zero exposure profile. However, the more complex versions of GUTS, and other prominent TKTD models which will be used for ERA in the near future remain untested.

This paper aims to prove the conditions under which unique multiplier values exist for the TKTD models described as ready for use or approaching readiness in EFSA’s Scientific Opinion on TKTD models^7^. The models in question are GUTS^4^, an example Dynamic Energy Budget (DEB) model^11^ and—in the supporting information (SI)—the primary producer models for microalgae^12^ and *Lemna*^13^. The proofs show under which circumstances $\hbox {EP}_{x}$s and similar quantities are guaranteed to exist and be unique. Each proof follows a similar philosophy, which should be applicable to other TKTD models. It is impossible to prove results from empirical experimental methods in the same rigorous way. However, our results inform experimental and model design, from both a scientific and regulatory perspective. Scientifically, any time accepted assumptions are shown to be invalid is an opportunity for discovery and improvement, either through model developments or new research to fill knowledge gaps. From a regulatory viewpoint there are implications for how to implement the EMF method. For instance, if uniqueness of the $\hbox {LP}_{x}$ cannot be guaranteed it is unwise to use a root-finding algorithm without some consideration of which $\hbox {LP}_{x}$—if any—is the right one to use in ERA.

## Materials and methods

### The exposure multiplication factor approach

It is possible for TKTD models to incorporate many different aspects to increase realism, including modelling different temperature^14^ and food availability, and mixtures of stressors^15^. However, there is currently no regulatory guidance on how to deal with the interaction of all of these sources of variability. Therefore, they are not currently included in TKTD models for standard ERA applications^7^. Instead, with the exception of the exposure concentration, all conditions in the model are typically fixed to the laboratory conditions maintained during the empirical bioassays. In essence, the model is simulating in silico versions of standard bioassays, but with many more exposure profiles than would be feasible in empirical, laboratory based experiments. We therefore refer to this strategy as a *laboratory mimic* approach. The approach limits complexity by focusing on a more thorough consideration of exposure compared to methods which produce single summary statistics.

Once a suitable TKTD model has been calibrated it is possible to run the model with any exposure profile and see the effects on the model organism relative to control conditions. For implementations within ERA, the model should also be properly validated according to the relevant guidelines^7^. An EMF scales the whole exposure profile according to that value. An EMF of 10 means that at any time the exposure in the model is 10 times greater than what is actually specified at that time in the unmodified exposure profile. The goal is to find an EMF value which imposes some specified percentage reduction in an aspect of organism performance. Typically, these will be the same as the measured outcomes in laboratory tests, for example survival probability or reductions in growth or fecundity.

The accepted use of GUTS in ERA finds the $\hbox {LP}_{x}$ value based on the survival probability at the end of the exposure profile. That is, $LP_{50}$ is the EMF which causes 50% toxicant induced mortality at the end of the exposure. We will extend the approach to the DEB-TKTD, algae and *Lemna* models, as is presumed by EFSA^7^. For sublethal effects, such as growth, reproduction or biomass, the $EP_{10}$ is the EMF which causes a 10% reduction in that quantity compared to control conditions in the model.

To find the $\hbox {LP}_{x}$ (or $\hbox {EP}_{x}$) an iterative algorithm tests different EMF values until exactly (within some numerical tolerance) $x\%$ effects occur at the end of the exposure profile. That EMF is the $\hbox {LP}_{x}$. For efficiency, a root-finding algorithm is often used. Over many iterations the algorithm should converge to the $\hbox {LP}_{x}$. However, if no $\hbox {LP}_{x}$ value exists, the algorithm could either run indefinitely or report the last tested EMF value before it stopped. In cases where multiple $\hbox {LP}_{x}$ values exist algorithms may converge to any of the different—but equally correct—$\hbox {LP}_{x}$ values (or produce an error).

In order to construct the proofs, we introduce new notation to signify a state variable which may change in response to the EMF. This is best done with an example. Consider survival, denoted by the state variable *S*(*t*). This is obviously dependent on time *t* but also, perhaps implicitly, dependent on the EMF, which we will denote $\alpha$. Survival when exposure has (potentially) been altered by the EMF $\alpha$ is denoted by $S(t; \alpha )$. As a result, survival under control conditions (i.e. zero exposure) is equivalent to *S*(*t*; 0) for any exposure profile, whereas the response to the unmodified exposure profile, i.e. prior to any multiplicative changes, is equivalent to *S*(*t*; 1).

### Proof methodology

For each model, the critical endpoint will be a change in one or more state variables in the model relative to the same variable under control conditions. Therefore we must show that a higher multiplier value applied to the exposure profile will always cause greater or equal effects on these state variables, a property known as monotonicity. Secondly, each variable affected by the EMF should be continuous with respect to the EMF, such that there are no values of the EMF which introduce discontinuities or undefined values in the state variables. Then, for some desired $x\%$ response, all that remains is to show that arbitrarily large EMFs cause greater than $x\%$ effects. When all these conditions are met, by the Intermediate Value Theorem (IVT) we know that some critical EMF leading to exactly $x\%$ exists. For uniqueness we also require strict monotonicity (without equality) in some neighbourhood of the critical EMF, otherwise the critical EMF will be some interval, rather than a single value.

Each of these proofs result in one or more theorems for the corresponding TKTD model. Only the theorems are presented in the main text. The details of each proof, and all model equations, are provided in the SI.

### Models

#### The GUTS framework

The GUTS framework is a set of nested models which quantify organism survival over time, typically under chemical stress^4^. In the full version of the model, uptake and elimination of the external concentration occurs according to a simple one compartment ODE which describes the internal concentration over time. Internal concentration then induces *damage*, another one-compartment model with accrual proportional to the internal concentration and repair proportional to the current damage. Each organism has some threshold for sensitivity drawn from a probability distribution, typically the log-logistic function. The individual’s probability of death increases linearly according to some rate parameter (the killing rate) multiplied by the extent to which damage exceeds the threshold.

**Figure 1:**
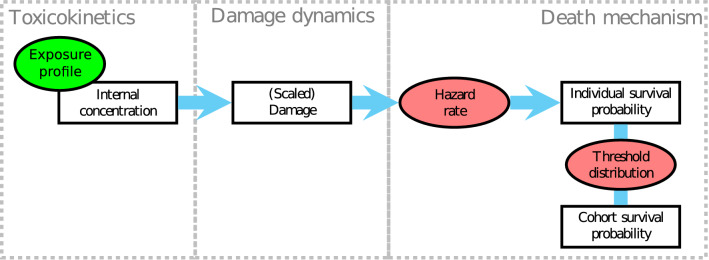
Schematic of the GUTS model framework. Boxes represent state variables within the model. Red ellipses are functions and the exposure profile is the forcing variable. Reduced forms (GUTS-RED) collapse the toxicokinetics and damage dynamics boxes into one state variable. The SD death mechanism assumes all organisms in the cohort have the same sensitivity threshold. The IT death mechanism assumes immediate death once the individual’s threshold is exceeded.

Typically, reduced forms of the model (referred to as GUTS-RED) are expected to be used in the ERA of PPPs^7,16^. These reduced models replace the internal concentration and damage ODEs with a single state variable, scaled damage, so called because it has the same dimensions as the external concentration^4^. As a further simplification, the death mechanism is simplified to one of two extreme cases. The first of these, stochastic death (GUTS-RED-SD), assumes that each organism has the same threshold value, once the scaled damage exceeds the threshold, death is a chance process. Secondly, the individual tolerance (GUTS-RED-IT) model takes the killing rate to infinity, such that death is instantaneous for the individual as soon as its threshold damage is exceeded. These simplifications enable GUTS-RED-SD and GUTS-RED-IT to be calibrated from standard mortality bioassays. A full schematic of the model, similar to that presented in^16^ is presented in Fig. 1.

The output of the model is the survival probability over time. The goal is therefore to find the EMF value which causes an $x\%$ reduction in the survival probability due to toxicant induced mortality at the end of the exposure profile.

#### DEB models

DEB also does not refer to a single model but rather a set of models which share many core assumptions. DEB theory uses a number of physical and biological laws to derive a mechanistic model of ODEs for the growth, reproduction and survival of an organism^17^. DEB-TKTD (sometimes referred to as DEBtox) models include additional equations to describe the uptake, transformation and elimination of a substance and the stress it causes. As such it can model the endpoints from standard aquatic animal bioassays and make predictions about untested exposures.

The full suite of DEB-TKTD models has recently been reviewed for their suitability for standardised ERA applications in the near future^18^. For this analysis we will use the recent reserve-less DEBkiss model of Jager^11^. We chose this model as it is a simple, comprehensive implementation for an animal’s post-birth lifecycle under some known exposure profile $C_w$. This simplicity reduces the amount of data required for model calibration. The derivation of the model is presented thoroughly in the original publication. A summary of the model and all state variables and model parameters are given in the SI. Here only a brief description is given.

All DEB models consider the assimilation, distribution and dissipation of energy within a single organism. In DEBkiss models, energy assimilated through food is immediately split between two branches. The somatic branch receives the fraction $\kappa$ of all assimilated energy. From this energy, somatic maintenance (proportional to the volume of the organism) is paid, and the remainder goes toward growth of new structure (biomass). This structure is tracked with the state variable structural length, *L*. The remaining $(1-\kappa )$ fraction is spent on maturation processes. Energy in this branch is first used to pay maturity maintenance (again linked to volume in this model) with the remainder being invested in maturation (for juveniles) or reproduction (adults). Here, the model of Jager^11^ makes two common simplifications to reduce model complexity. Firstly, we assume that size is a proxy for maturity, this means that maturation does not need to be modelled; the switch from juvenile to adult occurs once the animal has reached a given size. Secondly, the model assumes that reproduction is a continuous process rather than discrete. Although untrue, this assumption is useful for many species^19^. Cumulative reproduction $R_c$ is the second state variable.

When conditions worsen, for example due to increased exposure, it is possible that the organism will no longer have enough energy to pay somatic maintenance. This is starvation. Different species are likely to have different responses when this occurs. For our purposes, the model animal ceases growth and redirects sufficient energy from the reproductive branch to the somatic branch to pay somatic maintenance. If somatic maintenance costs exceed all energy assimilated from food then the animal must also shrink. It does this by burning structure to release energy with yield $y_P$, between zero and one. Structural length, *L*, decreases during this phase.

While different core DEB model variants may be used for numerous reasons, e.g. to better suit the model organism, substance or model environment, the TKTD elements differ little between these^18^. The best approach to utilising DEB-TKTD in ERA is therefore also not species specific. Traditionally, stress in DEB-TKTD models was directly linked to the (scaled) internal concentration^17,20^. However, experience with GUTS has shown that often toxic effects are not well described by the internal concentration^21^, and thus a scaled damage state variable is now proposed for DEB as well. The uptake and elimination rate of damage is governed by a dominant rate constant, $k_d$. Growth and reproduction processes can alter the uptake and elimination of damage, and thus may have an effect on damage dynamics. These influences on damage are known as “feedbacks”^11^. All feedback processes are represented as $x_i$, where the subscript changes to describe each process. If uptake or elimination occur across a membrane (e.g. the skin) which grows as the organism grows, then the surface area to volume ratio will affect the rate of either process. This ratio changes over time. These feedbacks are denoted $x_u$ and $x_e$ respectively. Additionally, damage can also be diluted, either by growth ($x_G$) or by reproduction ($x_R$). Each process may or may not be relevant for any given stressor, this is represented by a binary vector

1 $$\begin{aligned} \varvec{X} := [\varvec{X}_u, \varvec{X}_e, \varvec{X}_G, \varvec{X}_R]. \end{aligned}$$

Under this scheme, traditional DEB-TKTD models which used internal concentration, have $\varvec{X} = [1, 1, 1, 0]$. Suggestions on when each feedback is likely to be active ($\varvec{X}_i = 1$) or inactive ($\varvec{X}_i = 0$) are given by Jager^11^.

Above some tolerance threshold, damage creates stress, *s*, to the organism, which increases linearly, just as in the GUTS framework. This stress then perturbs one or more processes according to the physiological mode of action (pMoA) of the substance. Standard pMoAs are an inhibition to assimilation, $s_A$, increased maintenance costs, $s_M$, increased growth costs, $s_G$, increased reproduction costs, $s_R$ and hazard to the embryo during oogenesis, $s_H$. Stresses not active for a given pMoA are zero. We will sometimes refer to the pMoA as a binary vector $\varvec{S}$ such that

$$\begin{aligned}{}[s_A, s_M, s_G, s_{R}, s_{H}] = s \times \varvec{S}. \end{aligned}$$

**Figure 2:**
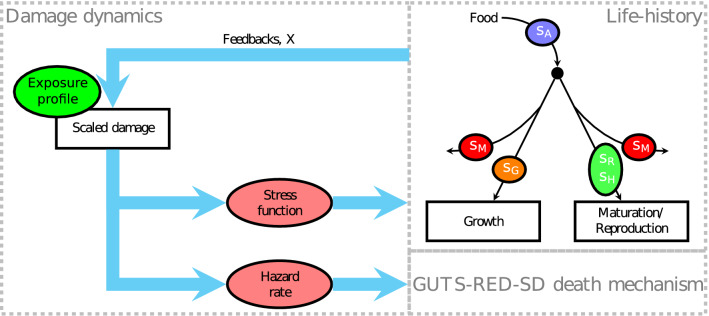
Schematic of the DEB-TKTD model. The exposure profile acts as a forcing variable on the scaled damage. The life-history diagram shows the energy fluxes within the organism. The stress function affects one or more processes marked by the ellipses. Damage also affects survival probability through the GUTS-SD death mechanism. Feedback processes (1) mean that growth and reproduction can alter the uptake and elimination of damage.

It is common to couple a DEB-TKTD model to a survival module following the GUTS-RED-SD framework^22,23^. We will also follow this approach to extend the utility of the results. The survival probability state variable is denoted by *S*. A full schematic of all elements of the model is given in Fig. 2.

Comparing the schematics in Figs. 1 and 2 shows the similarities in many elements of the model structures. For both models, the core elements of damage accumulation and repair are first-order processes. Indeed, if there are no feedback processes ($\varvec{X} = [0, 0, 0, 0]$) the survival probability over time predicted by the DEB-TKTD model will be identical to a GUTS-RED-SD model.

The extra complexity of the DEB model means that the model organism is no longer constant over time, it grows and reproduces. This introduces new state variables for these processes which can also be affected by stress, and investigated with the EMF approach. Additionally, the growth and reproduction processes can impact the damage uptake and elimination processes through the feedback processes. These processes are not included in a GUTS model, since there the model organism is not changing over time. For more information on GUTS and DEB models, see EFSA 2018^7^.

For this DEB model there are three model outputs which could be assessed with the EMF approach: reduction in size, fecundity or survival at the end of the exposure profile. Each of these relates directly to one of the state variables in the DEB model, *L*, $R_c$ or *S*, respectively.

## Results

### The GUTS model

For completeness, we will prove the result for the most general GUTS model, which will also prove the result for all reduced forms.

#### Theorem 1

*For any model version within the GUTS framework, let* $S(t; \alpha )$ *denote the survival probability at time* *t* *for a given non-zero exposure profile* $C_w(t)$ *scaled by some EMF value* $\alpha$. *For any chosen* $x> 0$ *percentage effect (exposure-induced mortality), model end time* $t_{E}$ *and background mortality* $h_b$ *low enough such that* $S(t_E; 0) > 0$ *there exists a unique EMF* $\alpha _*$ *such that*

2 $$\begin{aligned} S(t_{E}; \alpha _*) = \left( 1 - \frac{x}{100}\right) S(t_E; 0), \end{aligned}$$

$\alpha _*$ *is the* $\hbox {LP}_{x}$ *for the exposure profile.*

Baudrot and Charles^10^ calculated $LC_{50}$ values for GUTS-RED-SD and GUTS-RED-IT. Their results implied the result of Theorem 1 for the main regulatory models. Our work makes the result explicit and generalises it to the whole GUTS framework. Another result of Theorem 1 is that the $\hbox {LP}_{x}$ is monotonically increasing with respect to *x*. For example, the $\hbox {LP}_{50}$ will always larger than the $\hbox {LP}_{10}$ for the same exposure profile. This result comes directly from (S4) in the SI.

### DEB models

Due to the additional complexity of the DEB model we split the result into multiple theorems and proofs, starting by showing continuity and monotonicity of the damage ODE.

#### Theorem 2

*Let* $C_w$ *be some external concentration over time. Assume an effects model where the effects of higher exposure on growth and/or reproduction are always adverse (or zero) at all points in time. Then, defining the scaled damage ODE as*

3 $$\begin{aligned} \begin{aligned} \frac{D(t; \alpha )}{dt} =&k_d(x_u \alpha C_w - x_eD) - (x_G + x_R)D. \end{aligned} \end{aligned}$$

*Then, for any combination of feedbacks* $\varvec{X} = [\varvec{X}_u, 0, \varvec{X}_G, \varvec{X}_R]$, *damage is monotonically increasing with respect to* $\alpha$, *and is continuous with respect to* $\alpha$ *as long as changes to* *L* *and* *R* *are continuous. Moreover, damage is strictly monotonically increasing with respect to* $\alpha$ *whenever* $D(t;1) > 0$.

The limitation that $\varvec{X}_e = 0$ will be discussed in greater detail later. However, depending on the pMoA of the stressor we can extend the result of Theorem 2 slightly.

#### Corollary 3.1

*If the pMoA of a substance directly affects reproduction and does not affect growth, i.e.* $\varvec{S} = [0, 0, 0, s_{R}, s_{H}]$ *then the results of Theorem* 2*holds for any combination of feedbacks.*

Finally, we can step from the results of Theorem 2 and Corollary 3.1 to show the existence and uniqueness of a critical multiplier ($\hbox {EP}_{x}$ or $\hbox {LP}_{x}$) for growth, reproduction and survival.

#### Theorem 3

*Consider the DEB-TKTD model of Jager*^11^ *and a substance such that at least one of Theorem* 2*or Corollary* 3.1*hold. Further, let* $C_w(t)$ *be a non-zero exposure profile where the time of first exposure is before* $t_1$ *as defined in Table* 1. *Then, for any chosen percentage effect level* $x > 0$ *there exists a unique EMF* $\alpha _*>0$ *such that*

4 $$\begin{aligned} \min \left( \frac{L(t_{E}; \alpha _*)}{L_(t_{E}; 0)}, \frac{R_c(t_{E}; \alpha _*)}{R_c(t_{E};0)}, \frac{S(t_{E}; \alpha _*)}{S(t_{E}; 0)}\right) = 1 - \frac{x}{100} \end{aligned}$$

*this* $\alpha _*$ *is the* $\hbox {EP}_{x}$ *(or* $\hbox {LP}_{x}$) *for the exposure profile* $C_w(t)$.

**Table 1:** Table of the state variables and pMoAs (including combinations of pMoAs) in the DEB-TKTD model^11^.

Endpoint of concern	$\varvec{S}$, pMoA				
	[0, 0, 0, 0, 0]	$[0, 0, 0, s_R, s_H]^*$	$[0, 0, 1, s_R, s_H]$	$[1, 0, s_G, s_R, s_H]$	$[s_A, 1, s_G, s_R, s_H]$
Length, *L*	N/A	N/A	$L(t_1) < \frac{100 - x}{100}L(t_{E}; 0)$	$L(t_1) < \frac{100 - x}{100}e^{\frac{r_B}{y_P}(t_{E} - t_1)} L(t_{E}; 0)$	$t_1 < t_{E}$
Reproduction, $R_c$	N/A	$R_c(t_1) < \frac{100 - x}{100}R_c(t_{E}; 0)$			
Survival, *S*	$t_1 < t_{E}$				

In each case, $t_1$ is first time point where the external concentration is non-zero. Each entry in the table shows the condition which must be met in order for $x\%$ effects to be possible at the end of the exposure profile ($t_E$). N/A is used for pMoAs which do not affect the state variable. In cases where the condition is not met for a given variable, a multiplier may still be found for the others. Under assimilation stress (fourth column) there is a maximum rate at which length *L* can decrease. This rate is the von Bertalanffy growth rate, $r_B$, divided by the yield of burning structure to provide energy, $y_P$. *At least one of the two reproductive pMoAs must be active.

The monotonicity of effects on all state variables in the DEB model means that, for the conditions described in Theorem 3, the $\hbox {EP}_{x}$ (or $\hbox {LP}_{x}$) is also monotonically increasing with respect to *x*.

We should note here that one can either setup an algorithm to find the critical multiplier value for growth, reproduction and survival individually and then select the minimum or setup the algorithm to directly find the minimum critical multiplier as in (4). Both will produce the same result, but the second approach is likely to be faster.

One could argue that ERA should consider the combined effects of lethal and sublethal stress on the individual’s fitness. This is possible using the continuous form of the Euler–Lotka equation^24^

5 $$\begin{aligned} B(t) = \int _0^t B(t-a) l(a)b(a) da, \end{aligned}$$

where *B*(*t*) is the number of births at time *t*, *l*(*a*) is the fraction of females which survive to age *a* and *b*(*a*) is the birth rate for mothers of age *a*. For the offspring of a test population which all have the same age (as is the standard in long-term toxicity experiments) this integral collapses to a single point, $B(t-a) = 1$ when $t=a$ and zero elsewhere. The DEB model provides exactly the values which we need to calculate *B*(*t*). Namely

$$\begin{aligned} l(a) = S(a), \quad b(a) = \frac{d}{dt}R_c(a). \end{aligned}$$

One can now find the births per individual per time predicted by the DEB model as

6 $$\begin{aligned} B(t) = S(t)\frac{d}{dt}R_c(t). \end{aligned}$$

Integrating (6) over the duration of the experiment gives the expected number of offspring produced per female alive at the start of the test.

There are two clear options for how to proceed. Firstly, one could calculate $\int _0^{t_E} B(t) dt$ for each EMF and compare it to the control, similar to finding $\hbox {EP}_{x}$ values for individual endpoints. Alternatively, one can use *B*(*t*) as the basis to estimate the intrinsic population growth rate^25^. This quantity provides an estimation of population growth based on the survival and fecundity over time of individuals. Indeed, it is listed as a potential output value in the experimental guidelines for standard *Daphnia magna* reproduction tests^26^. For the first of these options we offer an extension to Theorem 3.

#### Corollary 3.2

*Consider a DEB-TKTD model and exposure profile such that Theorem* 3*holds. The number of expected offspring per female, given by*

$$\begin{aligned} \mathrm {B}(t_E; \alpha ) = \int _0^{t_E} S(t; \alpha )\frac{d}{dt}R_c(t; \alpha ) dt \end{aligned}$$

*has a unique* $\hbox {EP}_{x}$ $\alpha _*$ *such that*

$$\begin{aligned}\frac{\mathrm {B}(t_E; \alpha _*)}{\mathrm {B}(t_E; 0)} = 1 - \frac{x}{100}\end{aligned}$$

Our results provide a rigid boundary to the applicability domain of the EMF approach both in terms of existence and uniqueness. Existence relies on the initial time in the profile when external concentration is non-zero, as described in Table 1. While it is important to know about these conditions, they will rarely inhibit an ERA, since long initial periods with zero exposure are uncommon.

Cases where uniqueness cannot be guaranteed require more caution and it is unwise to use root-finding algorithms. In the next subsection we explore what can happen outside of this domain and provide suggestions for how to still produce a single reliable $\hbox {EP}_{x}$ value.

#### Surface:volume scaling of elimination

There is a reason that in Theorem 2, $\varvec{X}_e = 0$ was specified. In some cases when $\varvec{X}_e = 1$ a higher multiplier is no guarantee of higher damage for all time. Consider a substance which acts on assimilation and has surface area:volume scaled elimination (i.e. $\varvec{X} = [0, 1, 0, 0]$). The damage ODE under some EMF $\alpha$ is then

$$\begin{aligned} \frac{dD}{dt} = k_d \left( \alpha C_w - \frac{L_m}{L} D \right) , \end{aligned}$$

where $L_m$ is the maximum length the organism can reach. The EMF has a positive direct effect on damage, but also an opposing indirect effect. Increasing damage decreases the size of the organism which, due to the surface area:volume elimination of damage, enables faster elimination of damage. As a result, not only does Theorem 2 no longer hold but in fact a larger multiplier value can cause lower damage at some points in an exposure profile. In other words, we observe a paradoxical result whereby more exposure translates to less effect some time after exposure.

**Figure 3:**
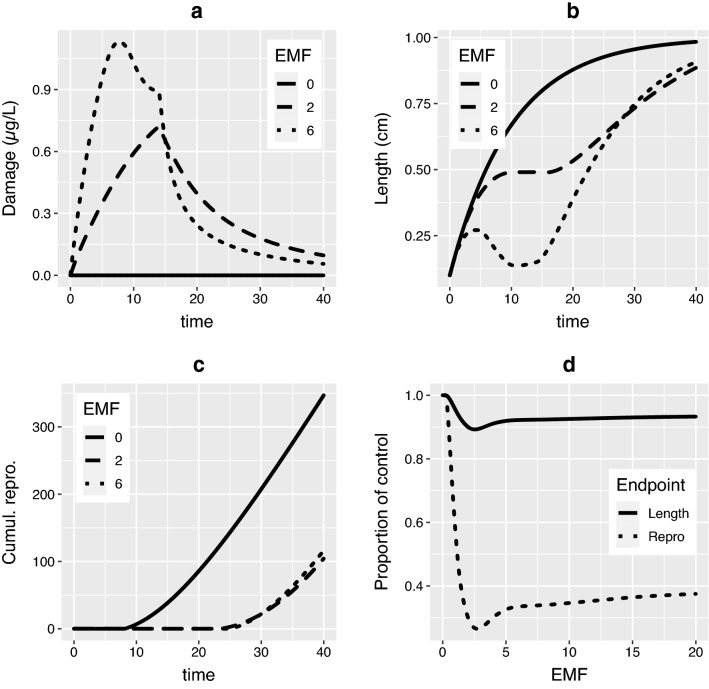
An illustration of the issues which can occur using the EMF approach for substances with surface area:volume scaled elimination (i.e. $\varvec{X} = [0, 1, 0, 0]$). The (non-multiplied) exposure is a constant $1 \mu g/L$ for the first 14 days and zero thereafter and effects assimilation only ($\varvec{S} = [1, 0,0, 0, 0]$). (**a**) Scaled damage, (**b**) length over time, (**c**) cumulative reproduction. (**d**) Endpoint value as a proportion of control after 40 days. The shape of these curves show that certain effect levels can be caused by two distinct multiplier values. Parameter values are $L_0 = 0.1$, $f = 1$, $r_B = 0.1$, $L_p = 0.6$, $L_m = 1$, $R_m = 15$, $\kappa = 0.8$, $y_P = 0.64$ $z_b = 0.1$, $b_b = 1$, $k_d = 0.05$, $\varvec{X} = [0, 1, 0, 0]$. See the SI for the definitions of these parameter values.

Figure 3 illustrates what we will refer to as the “more is less” scenario. The exposure consists of a single pulse early in the animal’s life, modelled for two multiplier values, $\alpha _2 > \alpha _1$. During the exposure phase the direct effect of the higher exposure causes higher damage and greater effects on size. After the pulse, external exposure is zero, and therefore the external concentration and uptake remain zero regardless of $\alpha$. Regardless of the EMF, scaled damage can only decrease during this phase. However, the effects of the higher multiplier are still relevant. As Fig. 2 shows, the feedback processes still influence damage dynamics. The model organism exposed to $\alpha _2C_w$ is smaller and therefore able to eliminate damage more rapidly because $\varvec{X}_e = 1$. This eventually leads to lower damage for the model organism exposed to $\alpha _2C_w$ (i.e. $D(t; \alpha _1) > D(t; \alpha _2)$). The more is less phenomenon can also impact growth and cumulative reproduction, as seen in Fig. 3b,c. Sometime after exposure $L(t;\alpha _2) > L(t; \alpha _1)$ and $R(t;\alpha _2) > R(t; \alpha _1)$. For survival, and any additional endpoints without recovery, this “crossover” is unlikely, mortality during the exposure phase (where $D(t; \alpha _2) > D(t; \alpha _1)$) will almost certainly dominate any mortality during the recovery phase. Figure 3d shows that for certain $x\%$ effect levels (vertical axis) multiple $\hbox {EP}_{x}$ values exist.

In practice, instances of non-uniqueness such as Fig. 3 will be rare since they rely on a sudden and significant decrease in external exposure. Moreover, EMF methods for DEB-TKTD models will include a moving time window method^18^ consisting of many exposures constructed sequentially and assessed. Each window will produce an $\hbox {EP}_{x}$ value, but only the lowest will be relevant for the ERA. A time window which starts slightly earlier in the broader exposure profile would feature the same pulse later in the model organism’s lifespan and thus not allow organism recovery. Depending on the exact endpoint used, one would expect those windows to have a lower (and unique) $\hbox {EP}_{x}$. However, the potential for multiple $\hbox {EP}_{x}$ values raises concerns across all areas which impose a multiplicative margin of safety. We cannot guarantee that a multiplier resulting in $x\%$ effects exists nor that any value found by the algorithm is unique.

Although not pictured here, maintenance and growth pMoAs and combinations of feedbacks which include $\varvec{X}_e = 1$ can also produce the “crossover” in the damage values and the “more is less” phenomenon seen in Fig. 3. It can also arise for scenarios which do not feature a deviation from the standard rules for growth (e.g. a starvation phase) and for other DEB based models. The SI features a similar plot to Fig. 3 showing damage crossover for a standard DEB model.

Knowing this, the obvious question is how to proceed? Certainly with caution when $\varvec{X}_e = 1$ is necessary in model calibration and validation. Under such circumstances algorithms must ensure that the $\hbox {EP}_{x}$ value found is the lowest multiplier which gives $x\%$ effects when there is a risk of non-uniqueness. The brute force approach, incrementing from zero until the desired effect level is met or exceeded, is one example. Whether it is realistic for higher EMF values to cause reduced effects in vivo then does not alter the conservatism of the approach for ERA.

Table 2 summarises the domain where the margin of safety approach can be used in conjunction with a root-finding algorithm without concern in the DEB-TKTD model of Jager^11^. For model configurations where non-uniqueness could emerge using another method to find the $\hbox {EP}_{x}$ is advisable. For example, a brute-force approach starting from an EMF of 0 in small increments (e.g. by 0.1). Without good reason, calibration should first be attempted with no feedbacks. Under this guiding philosophy of pursuing model simplicity we expect that the problem cases will be rare.

**Table 2:** A table to mark under which scenarios the EMF approach is and is not guaranteed to produce a unique $\hbox {EP}_{x}$.

	pMoA				
Feedbacks, $\varvec{X}$	Assim	Maintenance	Growth	Reproduction costs	Embryonic hazard
$[\varvec{X}_u, 1, \varvec{X}_G, \varvec{X}_R]$	Uniqueness not guaranteed			Corollary 3.1	
$[\varvec{X}_u, 0, \varvec{X}_G, \varvec{X}_R]$	Theorem 3				

Theorem/corollary references denote how uniqueness is assured. The $\varvec{X}_i$ terms denote that the corresponding theorem applies regardless of whether the feedback process is active or not. Note that when combinations of pMoAs are present, the most negative result holds. The problematic scenarios occur when $\varvec{X}_e = 1$ and the pMoA affects assimilation, maintenance or growth.

#### Other issues

The damage crossover illustrated in the previous subsection occurs more commonly, and to a greater extent, when the pMoA is assimilation effects. This is because, at least in this standard implementation, stress can cause $100\%$ effect and completely cease assimilation when $s_A \ge 1$ (see SI for details). When this is the case, higher exposure (even from an increased multiplier) does not translate to higher stress. This differs from other pMoAs, whose stress values are unbounded. Indeed, replacing $1 - s_A$ with $1/(1 + s_A)$ in the model ((S5) in the SI) reduces the occurrence and scale of “crossovers” such as Fig. 3. However, the formulation of the pMoA should not be based on how it might affect the algorithm or the EMF.

Certain species require further deviations from the standard model. For instance, different life-stages, growth and/or reproduction rules might be introduced to explain observed phenomena. Before models featuring these deviations are used in an EMF approach one should consider the potential issues as we have done in this section. While a proof of existence and uniqueness of the $\hbox {EP}_{x}$ for each model variant is ideal it is also infeasible. However, modellers should ensure that their approach is robust enough to deal with issues around existence and uniqueness. Checking that the model endpoint is reduced by $x\%$ when the $\hbox {EP}_{x}$ is applied to the exposure profile is an easy way to check accuracy and existence. An argument (if not a full, formal proof) for uniqueness should also be considered. In cases where that is not possible, the algorithm must be set up to identify the lowest $\hbox {EP}_{x}$, or check that no lower values exist.

One common addition is to DEB-TKTD models which feature starvation is to assume that there is some maximum amount of starvation/shrinking which an animal can survive. Once that point is met or exceeded death is instantaneous^27^. Such death mechanisms cause problems. They can introduce a discontinuity in the response versus multiplier value for a given time window (i.e. a “jump” in plots such as Fig. 3). For instance, if in the example given in Fig. 3 the animal was not allowed to shrink, and instead died, then the multiplier of 6 would result in $100\%$ effects on survival (and significant growth effects). In contrast, the exposure when the multiplier is 2 is survivable and the animal can recover. Presumably, for some critical $\alpha _c \in (2, 6)$ the exact threshold for death is reached. This $\alpha _c$ is a discontinuity between partial and $100\%$ effects relative to control. Under some circumstances this will prohibit finding a multiplier which results in exactly $x\%$ effects, regardless of the method used.

There are two readily apparent solutions to this at the individual level. One is to set $\alpha _c$ as the multiplier for the window, the second is to replace such discrete responses with graded responses. In this example for instance, shrinking could add to the lethal hazard *h*. It is not possible to universally recommend one approach over the other as it will depend on the species’ behaviour. Once that decision has been made these issues must be recognised and reported by the modellers.

## Discussion

Using mechanistic models to better understand and predict the effects of exposure to potentially harmful substances such as PPPs is clearly of great value. Especially for aquatic organisms, where we often have the luxury of a well defined predicted exposure, the path ahead may seem obvious. However, as we have shown, implementing models—especially for sublethal effects—requires care.

In most previous work, it has simply been assumed that the EMF approach will produce a unique value for any level of effects relative to the control. This is not the case. By pursuing rigorous mathematical proofs we have found certain restrictions and exceptions. For the GUTS model framework, any non-zero exposure profile has a unique $\hbox {LP}_{x}$ for any *x*. For the primary producer models, the major findings are the restrictions on the exposure profile. Since both the algae and *Lemna* models have some maximum rate of biomass or population decay, the first instance of non-zero external concentration in the exposure profile must occur with sufficient time before the end of the profile for the effect of the substance to exceed $x\%$. For more information see the SI. Similar findings occur for some of the pMoAs in the DEB model, as shown in Table 1. The reason behind this is the same as for the primary producer models.

When using these models to make predictions it is important to realise that we must protect organisms (or populations in the case of algae) of different ages and sizes. For GUTS models this has no effect, but for DEB the time that exposure occurs within its lifespan is important. For instance, effects of exposure to a substance which increases growth costs will have more effect on a juvenile, growing organism than a fully grown adult. For this reason, moving time-windows have been suggested in ERA. The method constructs consecutive intervals of time from the exposure profile of concern and finds the $\hbox {EP}_{x}$ for each window. The $\hbox {EP}_{x}$ for the profile as a whole is the minimum of these values^18^. The approach also guarantees that some windows will have immediate exposure. This means that, although non-existence could occur for certain windows, a relevant $\hbox {EP}_{x}$ for the exposure profile will always exist. These results provide confidence in the EMF method and the use of the most efficient root-finding algorithms to calculate $\hbox {LP}_{x}$ and $\hbox {EP}_{x}$ values for these models.

However, for DEB models there are further limitations due to the possible feedbacks and model extensions. The feedback options in (1) are important to accurately describe damage dynamics in a one-compartment module. Finding the appropriate feedback mechanisms can significantly improve the correspondence between the model and the data^11^. Until recently the configuration $\varvec{X} = [1, 1, 1, 0]$ was the default in DEB-TKTD applications^17,22^. This meant that every model application featured the problematic surface area:volume scaling of elimination. Although these feedbacks match our intuition and knowledge for dermal uptake and elimination the “more is less” multiplier paradox is a non-intuitive result which can arise in this scenario and indeed any other scenario with $\varvec{X}_e = 1$. However, as was shown in Theorem 3 and Corollary 3.1, uniqueness is guaranteed for all reproductive pMoAs, and substances with any pMoA as long as $\varvec{X}_e = 0$.

Whether the behaviour illustrated in Fig. 3 can occur in real-world tests is unknown. Experimental data exploring this would be of value regardless of the results. The majority of ecotoxicology, especially ERA, is built on the presumed idea that scaling (i.e. multiplying) exposure will scale the effects in the same direction. Using the well-known and highly respected DEB theory we have shown that this might not always be the case. If the behaviour in the model were to be observed experimentally it could change our perspective on ranking different exposures in terms of effects, at least at the individual level. On the other hand, if it was clearly refuted it could signal a need to alter and improve the TKTD aspects of the model.

The issues raised in this work are therefore important. It is imperative that modellers and anyone using the models are aware of the domain of applicability and demonstrate that they are within them. This applies to all TKTD models. The simplest solution to non-uniqueness in a regulatory context is to avoid root-finding algorithms in favour of (for example) a robust brute-force approach checking multiplier values from zero and increasing in small increments (e.g. 0.1) to approximate the lowest $\hbox {EP}_{x}$. Accuracy and conservatism are thus maintained by sacrificing elegance and computational efficiency.

The EMF approach of replicating standard in vivo experiments in silico is appealing. Models are used to generate the same endpoints that are recorded at the end of real-world experiments specifically designed and performed for the ERA of PPPs. However, taking proportional effects at the end of the in silico experiment is only one option. We have shown in Corollary 3.2 one example of how a new endpoint can be derived from model state variables and used for ERA. Alternatively, one could define the endpoint for ERA as the maximum proportional effect at any point in the window. Although we cannot a priori determine at what time the maximum effects will be observed, the results of Theorems 1 and 3 as well as Theorem S1 and Theorem S2 in the SI hold for any value of *t* and so will also guarantee uniqueness and existence of $\alpha _*$ as long as the time at which the difference is assessed remains constant for all EMFs. However, this approach is generally not recommended. To give one example, if reproduction is delayed at all compared to the control, during that delay there is a 100% reduction in reproduction compared to the control.

This work has capitalised on a hitherto under-utilised aspect of TKTD models: the ability to provide rigorous proofs of certain properties. By doing so, we have conclusively shown under which circumstances $\hbox {LP}_{x}$ and $\hbox {EP}_{x}$ values exist and are unique. These model results also lend some verification to the use of multiplicative AFs applied to standard bioassay results. It may not always be possible to prove similar results for other TKTD models. Nonetheless, it is essential to consider all possible results and behaviours of a model before using it in any official context.

## Supplementary Information

Supplementary Information.
