# Supplementary material for: The application and limitations of exposure multiplication factors in sublethal effect modelling
Source: Sci Rep. 2022 Apr 11;12:6031. doi: 10.1038/s41598-022-09907-1 (PMC9001712; doi:10.1038/s41598-022-09907-1)
Supplement: Supplementary file 1 — Supplementary Information. [file 41598_2022_9907_MOESM1_ESM.pdf]

Supplemental Material - The application and limitations of exposure multiplication factors in sublethal effect modelling

Neil Sherborne[1*], Tjalling Jager[2], Benoit Goussen[3], Marie Trijau[3], and Roman Ashauer[4,5]

[1]Syngenta, Jealott's Hill International Research Centre, Bracknell, Berkshire, RG42 6EY, UK. * Neil.Sherborne@Syngenta.com

[2]DEBtox Research, Stevensweert, The Netherlands

[3]Ibacon GmbH, Roßdorf, Germany

[4]Syngenta Crop Protection AG, 4058, Basel, Switzerland

[5]Department of Environment and Geography, University of York, Wentworth Way, Heslington, York, YO10 5NG, UK

S1 The GUTS model

We use the most comprehensive version of the GUTS model.[1] The full set of model equations is

$$\begin{aligned}
\frac{dC_i}{dt} &= k_u C_w - k_e C_i \\
\frac{dD_i}{dt} &= k_r (C_i - D_i) \\
h_z &= b_i \max(0, D_i - z_i) + h_b \\
\frac{dS_z}{dt} &= -h_z S_z \\
S(t) &= \int_0^\infty S_z(t) f(z_i; m_i, \beta) dz_i.
\end{aligned} \tag{S1}$$

The full derivation of the model is given by Jager and Ashauer.[1] Briefly, the uptake rate k_u determines how rapidly the external concentration C_w is taken up as an internal concentration, C_i. The elimination rate k_e determines the speed of the reverse process. This internal concentration leads to damage. Due to the difficulty of measuring damage we work instead with scaled damage, which re-frames damage with the same units as internal concentration. This scaling means that accrual and repair of damage occurs with the same rate parameter k_r.

Damage increases the lethal hazard on the organism. Each organism has some threshold z_i. Below this threshold damage has no effect on lethal hazard h_z. Once the threshold is exceeded hazard increases linearly with gradient b_i. There may also be a background hazard h_b which is intended to model accidental deaths, not deaths due to ageing. The hazard affects survival probability for the individual S_z as an exponential decay. To move from the individual to the test population survival probability over time we must integrate over the probability distribution of the threshold f. Typically, this is a log-logistic function with median m_i and slope parameter β.

S1.1 Proof of Theorem 1

First, we show that the critical EMF α_* exists. Clearly, when $\alpha = 0$ there are no effects. If we can prove that there exists some α_m such that $S(t_E; \alpha_m) < (1 - \frac{x}{100}) S(t_E; 0)$ and that $S(t; \alpha)$ is continuous with respect to α then by the intermediate value theorem α_* must exist.

For the internal concentration ordinary differential equation (ODE) we have the solution

$$C_i(t; \alpha) = e^{-k_e t} k_u \alpha \left[C_i(0; 1) + \int_0^t e^{k_e \tau} C_w(\tau) d\tau \right] = \alpha C_i(t; 1).$$

Where $C_i(0)$ is the initial condition for the internal concentration for any α, typically $C_i(0) = 0$. A similar result exists for the scaled damage, such that there is a linear relationship (i.e. $D_i(t; \alpha) = \alpha D_i(t; 1)$). In some ways this is an extension of the results of Baudrot and Charles,[2] who deal directly with the reduced form of GUTS. Thus one

can easily see that damage over time, D, is continuous and monotonically increasing with respect to α. The same therefore applies to the stress equation:

$$h_z(t;\alpha) = b_i \max(\alpha D_i - z_i, 0), \tag{S2}$$

since the maximum of continuous functions is itself continuous. Hazard is monotonically increasing with respect to α, and strictly monotonic if $\alpha D_i > z_i$. Then for survival we have

$$S(t_E;\alpha) = \int_0^\infty \exp\left(-\int_0^{t_E} h_z(\tau;\alpha)\,d\tau\right) f(z_i)dz_i, \tag{S3}$$

Equation (S3) is again continuous with respect to α. From (S2) and (S3) one can see that $S(t_E,\alpha) \xrightarrow{\alpha\to\infty} 0$ and thus a sufficiently large α_m can be chosen such that, by the intermediate value theorem some α_* exists in the interval $(0,\alpha_m)$.

It remains to prove that α_* is unique. To do this we must show that $S(t;\alpha)$ is monotonically decreasing with respect to α and *strictly* monotonically decreasing in some neighbourhood of α_*.

Consider some α_1 and α_2 where $\alpha_2 > \alpha_1 > 0$. From (S2) one can see that $h(t;\alpha_2) \geq h(t;\alpha_1)$ with equality if and only if $h(t;\alpha_2) = 0$. This also means that

$$S(t_E,\alpha_2) \leq S(t_E,\alpha_1) \tag{S4}$$

with equality if and only if $h(t;\alpha_2) = 0\ \forall t \in [0,t_E]$. This equality is impossible if α_1 is in the neighbourhood of α_* since some stress must occur during exposure in order to cause lethal effects. Thus $S(t;\alpha)$ is everywhere a monotonically decreasing function of α and strictly monotonically decreasing in some neighbourhood of α_*. Thus α_* is unique.

For the reduced model, the ODEs for internal concentration and scaled damage are replaced by a single scaled damage ODE with one rate parameter, the dominant rate constant k_d. Mathematically, this is equivalent to setting $k_u = k_e \to \infty$ and renaming k_r as k_d. The proof therefore holds for GUTS-RED. The two extreme death mechanisms are also covered by this proof as special cases of GUTS-RED for specific parameter values. □

S2 The DEB model

The model equations are

$$\begin{aligned}
\frac{dD}{dt} =& k_d(x_u C_w - x_e D) - (x_G + x_R)D \\
\frac{dL}{dt} =& r_B \frac{1+s_M}{1+s_G}\left(f L_m \frac{1-s_A}{1+s_M} - L\right) \\
R =& \begin{cases} \max\left(0, \frac{R_m}{1+s_R} e^{-s_H} \frac{f_R L_m L^2(1-s_A) - L_p^3(1+s_M)}{L_m^3 - L_p^3}\right) & \text{if} \quad L \geq L_p \\ 0 & \text{otherwise} \end{cases} \\
\frac{dR_c}{dt} =& R \\
\frac{dS}{dt} =& -(h+h_b)S
\end{aligned} \tag{S5}$$

The term f_R is the virtual food level for reproduction, used to model how much energy reproduction receives.[3] When the organism can assimilate sufficient energy to grow $f_R = f$. However, under starvation the model assumes that energy is first redirected from the reproductive branch to cover somatic maintenance costs and maintain structural length. When dL/dt becomes negative in (S5) f_R deviates from f to represent the proportion of energy still attributed to reproduction after maintenance costs have been paid. This strategy gives f_R as

$$f_R = \frac{f - \kappa \frac{L}{L_m}\frac{1+s_M}{1-s_A}}{1-\kappa} \tag{S6}$$

If $0 \leq f_R < f$ then this shifted energy flux is enough to pay somatic maintenance and $dL/dt = 0$ is enforced. When $f_R < 0$ this energy is no longer sufficient to maintain the animal's current size, and it must burn structure to pay the essential somatic maintenance costs. Here, a new parameter appears, the energy recovered from burning structure as

a fraction of the total energy initially invested in building that structure, y_P, otherwise the structure of the growth equation appears similar to that in the core model (S5)

$$\frac{dL}{dt} = \frac{r_B}{y_P}(1+s_M)\left(\frac{fL_m}{\kappa}\frac{1-s_A}{1+s_M} - L\right). \tag{S7}$$

Each x_i denotes a feedback mechanism in the TKTD processes. The first two, x_u and x_e respectively denote damage uptake and elimination changing with the ratio of surface area:volume. The remainders handle damage dilution, either through growth (x_G) or reproduction (x_R). Depending on the nature of the substance, the route of exposure, and the exposed species these processes may or may not be relevant. Entries of the 4×1 vector $\boldsymbol{X}$ act as switches for each of the feedbacks. Jager[3] derives these feedbacks as

$$\begin{aligned} \boldsymbol{X} &:= [\boldsymbol{X}_u, \boldsymbol{X}_e, \boldsymbol{X}_G, \boldsymbol{X}_R], \\ [x_u, x_e, x_G, x_R] &= \left[\max\left(1, \boldsymbol{X}_u\frac{L_m}{L}\right), \max\left(1, \boldsymbol{X}_e\frac{L_m}{L}\right), \boldsymbol{X}_G\frac{3}{L}\frac{dL}{dt}, \boldsymbol{X}_R\frac{dR}{dt}F_{BV}K_{RV}\right] \end{aligned} \tag{S8}$$

Each $\boldsymbol{X}_i$ takes the value of 1 when the feedback is active, and 0 when inactive.

Similarly, each s_i term represents a different form of stress caused by the scaled damage, D. Above some threshold z_b, damage causes stress to one or more physiological processes according to the physiological mode of action (pMoA) of the stressor. The standard pMoAs are: s_A decreased assimilation, s_M increased maintenance costs, s_G increased growth costs, s_R increased reproduction costs and s_H embryonic hazard during oogenesis.[4,5] Note that Jager[3] discounted embryonic hazard from their model. For the purpose of generality we have reinstated it in (S5). Damage translates to stress via the following equations

$$s = b_b \max(0, D - z_b) \quad h = b_s \max(0, D - z_s) \tag{S9}$$

where s is sublethal stress and h is lethal stress (or hazard). Each s_i term then takes on the value of s - if the substance exhibits that pMoA - or 0 if not (note that we restrict $s_A = \min(1, s)$). Similarly to the feedbacks, we will sometimes refer to the pMoA as a binary vector $\boldsymbol{S}$ such that

$$[s_A, s_M, s_G, s_R, s_H] = s \times \boldsymbol{S}.$$

S2.1 Proof of Theorem 2

For $\boldsymbol{X} = [0,0,0,0]$ the damage ODE is equivalent to that in the GUTS-RED models, and so $D(t;\alpha) = \alpha D(t;1)$, as was shown by Baudrot and Charles.[2] We must show that for each feedback the impact of turning them on (setting $\boldsymbol{X}_i = 1$) does not introduce any discontinuities nor affect monotonicity.

We examine each of the feedback processes individually, starting with $\boldsymbol{X}_u = 1$. If the substance reduces growth then the animal remains smaller compared to the control and thus, due to the structure of the damage ODE, it more rapidly accrues damage, leading to a superlinear relationship and retaining montonicity of damage.

The dilution terms, respectively active when $\boldsymbol{X}_G$ and $\boldsymbol{X}_R$ are one, both subtract from damage accrual. Dilution by reproduction is suppressed whenever the reproduction rate is suppressed. By assumption, higher damage always reduces the reproduction (this will be verified in Theorem 3) thus dilution is reduced. In other words, an increase in the multiplier not only increases damage uptake but also reduces elimination, thus causing a superlinear increase in $D(t;\alpha)$ and maintaining continuity and monotonicity of damage with respect to α.

Dilution by growth is more complex

$$x_G = \boldsymbol{X}_G\frac{3}{L}\frac{dL}{dt},$$

note that $\frac{1}{L}\frac{dL}{dt} = \frac{d}{dt}[\ln L]$ is the growth rate. Under some initial exposure, when D is increasing, or constant, then the growth rate is further suppressed (or unchanged) under higher multipliers, dilution by growth is reduced and the monotonicity argument from the previous paragraph holds. However, if the external concentration then decreases, the smaller animal may have an advantage over the larger animal as it has more scope to grow, and thus greater potential for dilution by growth. Given that we enforce $\boldsymbol{X}_e = 0$, the extent of this advantage is maximised when $\boldsymbol{X} = [0,0,1,0]$ and external exposure drops immediately to zero. After an initial phase of exposure the external concentration drops to zero at time $t = t_c$. From t_c onwards damage is repaired according to the ODE

$$\frac{dD(t;\alpha)}{dt} = -k_d D - 3\frac{d\ln L}{dt}D. \tag{S10}$$

This ODE has the solution

$$D(t;\alpha) = D(t_c;\alpha)e^{-k_d(t-t_c)}\exp\left[-3\int_{t_c}^{t}\frac{d}{d\tau}\ln L d\tau\right] \tag{S11}$$
$$= D(t_c;\alpha)e^{-k_d(t-t_c)}\exp\left[-3(\ln L(t;\alpha) - \ln L(t_c;\alpha))\right] \tag{S12}$$
$$= D(t_c;\alpha)e^{-k_d(t-t_c)}L(t_c;\alpha)^3 L(t;\alpha)^{-3} \tag{S13}$$

Now consider two model organisms o_1 and o_2 exposed to the same exposure profile under different multiplying factors, $\alpha_1 < \alpha_2$. At time t_c, $D(t_c;\alpha_1) < D(t_c;\alpha_2)$ and $L(t_c;\alpha_1) > L(t_c;\alpha_2)$. To see what happens for $t > t_c$ we can examine the ratio of the damage equations (S13) for both organisms over time

$$\frac{D(t;\alpha_2)}{D(t;\alpha_1)} = \frac{D(t_c;\alpha_2)}{D(t_c;\alpha_1)}\frac{e^{-k_d(t-t_c)}}{e^{-k_d(t-t_c)}}\frac{L(t_c;\alpha_2)^3}{L(t_c;\alpha_1)^3}\frac{L(t;\alpha_1)^3}{L(t;\alpha_2)^3} \tag{S14}$$

The first three fractions are constant, and we know that (S14) is greater than 1 for $t = t_c$. Therefore, the only way that the ratio can become less than or equal to one is if $L(t;\alpha_2) > L(t;\alpha_1)$. However, dilution by growth cannot cause this. If $L(t;\alpha_2)$ approaches $L(t;\alpha_1)$ then the dilution by growth processes tend to the same rate for both model organisms, o_1 and o_2. Hence sizes cannot cross-over, and therefore $D(t;\alpha)$ is monotonically increasing with respect to α.

Regardless of the combination of feedbacks, the only direct influence of α remains the same as in the proof of Theorem 1. Thus, discontinuities with respect to α can only come from discontinuities in L or R, which must be checked in the context of the chosen effect model. □

S2.2 Proof of Corollary 2.1

Since for these pMoAs growth is unaffected $L(t;\alpha) = L(t;0) =: L_c$ for any multiplier α. Hence the damage ODE becomes

$$\frac{dD(t;\alpha)}{dt} = k_d\left(\frac{\alpha C_w L_m}{L_m - \boldsymbol{X}_u(L_m - L_c)} - \frac{DL_m}{L_m - \boldsymbol{X}_e(L_m - L_c)}\right) - \boldsymbol{X}_G\frac{3}{L_c}\frac{dL_c}{dt}D - \boldsymbol{X}_R F_{bv}K_{Rv}\frac{dR}{dt}D,$$

where L_c denotes length under control conditions. Thus, as when $\boldsymbol{X}_e = 0$, the effect of the multiplier on the elimination term acts only through D, maintaining monotonicity. For instance, when $\boldsymbol{X} = [0, 1, 0, 0]$

$$D(t;\alpha) = e^{-k_d L_m \int_0^t L(\tau;0)^{-1}d\tau}\left[D(0;1) + \alpha k_d\int_0^t C_w e^{k_d L_m \int_0^\tau L(z;0)^{-1}dz}d\tau\right] = \alpha D(t;1)$$

□

S2.3 Proof of Theorem 3

From Theorem 2 we know that damage at any time is monotonically increasing with respect to the multiplier α. Hence the stress terms are also monotonically increasing with respect to the multiplier, i.e. for $\alpha_2 > \alpha_1$ we have $s_i(\alpha_2) \geq s_i(\alpha_1)$ for any time t.

Therefore, we must prove that

1. L, R_c and S are all continuous and monotonically decreasing (strictly in some region of α_*) with respect to the stress values.
2. As $\alpha \to \infty$ the effect level for at least one endpoint will exceed $x\%$

For survival, this proof matches that in Theorem 1, with possible changes in the damage ODE covered by Theorem 2 or Corollary 2.1 (with the caveat that sublethal effects must not introduce discontinuities). For the other endpoints, the situation is more complicated. Analytical solutions exist for these equations under control conditions[6] and integral equations can generalise these to variable exposure conditions.

For body length, under conditions which allow for positive growth, the integrating factor $e^{r_B\int_0^t\left(\frac{1+s_M}{1+s_G}\right)d\tau}$ gives

$$L(t) = e^{-r_B\int_0^t\frac{1+s_M}{1+s_G}d\tau}\left(r_B f L_m\int_0^t\frac{(1-s_A)}{1+s_G}e^{r_B\int_0^\tau\frac{1+s_M}{1+s_G}dz}d\tau + L_0\right). \tag{S15}$$

It is clear to see that L is continuous with respect to all stress terms. Observation of (S15) is enough to know that higher assimilation stress has a negative effect on $L(t)\ \forall t$. For simplicity, we can now set $s_A = 0$ to focus on s_M and s_G. Since s_M and s_G appear in multiple places in (S15) their overall impact is less intuitive. First, consider s_M in isolation, i.e. take $s_G = 0$, such that:

$$L(t) = e^{-r_B \int_0^t 1+s_M\, d\tau} \left(r_B f L_m \int_0^t e^{r_B \int_0^\tau 1+s_M\, dz} d\tau + L_0 \right). \tag{S16}$$

If s_M increases via an increasing multiplier, s_M grows (or remains zero) for all t, therefore the exponential term outside the main bracket is smaller for larger multipliers. However, it is not clear that increasing s_M will decrease size, since s_M appears in a positive and negative exponent. We want to show that the negative exponential term dominates the positive one, such that increasing s_M decreases $L(t)$.

Unfortunately, there is no closed solution of the integral in (S16). To proceed, we use the known integral

$$\int f'(y) e^{f(y)} dy = e^{f(y)}, \tag{S17}$$

letting $y := s_M$ and $f(y) := r_B \int_0^z (1+s_M) dz$, meaning that $f'(y) = r_B(1+s_M)$. Then the integral term within (S16) becomes

$$\begin{aligned} r_B f L_m \int_0^t e^{r_B \int_0^\tau 1+s_M\, dz} d\tau &= r_B f L_m \int_0^t \frac{r_B(1+s_M)}{r_B(1+s_M)} e^{r_B \int_0^\tau 1+s_M\, dz} d\tau & \text{(S18)} \\ &= f L_m \int_0^t \frac{f'(y)}{(1+s_M)} e^{f(y)\, dz} d\tau & \text{(S19)} \\ &\leq f L_m e^{f(y)} = e^{r_B \int_0^\tau 1+s_M\, dz} & \text{(S20)} \end{aligned}$$

where the inequality is formed since $s_M \geq 0$. Therefore the negative exponential dominates the one inside the bracket and, since L_0 is a constant, increasing s_M via a higher multiplier decreases $L(t)$ for all t.

For growth effects only, the integral within the main bracket of (S15) is naturally of the form (S17). Hence we have

$$\begin{aligned} L(t) &= e^{-r_B \int_0^t \frac{1}{1+s_G} d\tau} \left(r_B f L_m \int_0^t \frac{1}{1+s_G} e^{r_B \int_0^x \frac{1}{1+s_G} dx} d\tau + L_0 \right) \\ &= e^{-r_B \int_0^t \frac{1}{1+s_G} d\tau} \left(f L_m \left[e^{r_B \int_0^t \frac{1}{1+s_G} d\tau} \right]_0^t + L_0 \right) \\ &= \left(f L_m \left[1 - e^{-r_B \int_0^t \frac{1}{1+s_G} d\tau} \right] + L_0 e^{-r_B \int_0^t \frac{1}{1+s_G} d\tau} \right). \end{aligned}$$

Which again is monotonically decreasing with respect to the multiplier. Interestingly, this result means that when $\boldsymbol{X} = [0, 0, 0, 0]$ and the pMoA is only growth effects there may be a closed analytical solution for the whole DEB-TKTD model. More importantly, we note that combinations of these stress terms exacerbate effects on growth, meaning that higher multiplier values have a negative effect on $L(t)$ for any combination of assimilation, maintenance and growth stresses.

Given the similarity of the growth equation under shrinking, this argument can largely be repeated to show that higher multipliers enforce more rapid shrinking, retaining strict negative monotonicity.

It now just remains to ensure that the transitions between these points maintain these qualities. Note that to initiate starvation under ideal feeding conditions the pMoA must be either assimilation and/or maintenance. Exactly zero growth naturally occurs when $dL/dt = 0$ in the model (S5). For model organisms which cannot redirect energy from the reproductive branch one can consider this a threshold for shrinking, and rewrite it in the form:

$$L^* = f L_m \frac{1 - s_A}{1 + s_M}. \tag{S21}$$

For organisms which can redirect energy from the reproductive branch zero growth can be maintained over a range of conditions. This is modelled through the f_R term (4). For these organisms, shrinking occurs only when $f_R \leq 0$, which again is continuous and monotonically decreasing with respect to the stress terms. The critical point for shrinking is then

$$L^* = f \frac{L_m}{\kappa} \frac{1 - s_A}{1 + s_M}. \tag{S22}$$

In either case, the thresholds are variable in time and continuous and strictly monotonically decreasing with respect to both stress terms. These thresholds are such that if $L < L^*$ the organism can grow but if $L > L^*$ it must shrink.

Consider two model organisms, o_1 exposed to multiplier α_1, and o_2 exposed to $\alpha_2 > \alpha_1$. We denote L_i and L_i^* as the length and threshold length for organism i. It is difficult to prove that o_2 is shrinking at any time o_1 is shrinking. However, we can see that the threshold for shrinking is lower, i.e. $L_1^* > L_2^*$. If o_1 is shrinking but o_2 is not, then, by definition

$$L_1 > L_1^* \geq L_2^* \geq L_2.$$

Thus we have shown that continuity and monotonicity are retained even during transitions between growth and starvation. The arguments presented here for strict monotonicity of growth with respect to the multiplier for positive, zero and negative growth can be combined, so that even under exposures which cause multiple instances of each we can be sure that higher multipliers cause increased effects.

Continuity with respect to the multiplier is also guaranteed, all stress terms are continuous and transitions between growth phases are defined to be continuous (e.g. L^* is continuous with respect to the stress terms).

Fortunately, it is much easier to prove continuity and monotonicity for the reproduction equation. We assume some amount of reproduction in the control. Since growth has already been proven to be continuous and monotonically decreasing with respect to the multiplier careful examination of the reproduction equation (in particular the stress terms and $L(t)$) is enough to see that reproduction also fulfils these requirements. Within f_R (S6) there is a theoretical concern of the asymptote at $s_A = 1$. However, this point lies beyond the region where shrinking begins and zero reproduction is enforced by the maximum function in (S5).

Unlike growth, the rate of reproduction at any given time cannot exceed the rate under control conditions, as long as length does not exceed length in the control. Therefore any reduction in dR_c/dt is still evident at the end of the time window. Hence it is easy to see that cumulative reproduction is also monotonically decreasing with respect to the multiplier and, in the region of the critical multiplier for reproduction, this is a strict monotonicity by the same argument as the proof of Theorem 1.

Finally, as $\alpha \to \infty$ the negative effects of exposure increase. Even if there is a period of zero exposure where the animal may be able to recover, higher damage will take longer to eliminate, postponing the recovery of growth and/or reproduction. As long as non-zero exposure occurs early in the profile, large effects are possible. However, it will sometimes be impossible to reach 100% effects. Some maximal effect level below 100% occurs if the first exposure comes late in the time window. Let t_1 be the first time that $C_w(t;\alpha) > 0$. For survival, taking $\alpha \to \infty$ causes more rapid onset of effects once exposure is non-zero, so as long as $t_1 < t_E$ any x% effects can be found. For reproduction, maximum effect is zero additional reproduction, therefore t_1 must be such that

$$R_c(t_1;\alpha) < \frac{100-x}{100} R_c(t_E;0) \tag{S23}$$

so that an immediate and complete stop to reproduction has x% effects when $t = t_E$.

The growth endpoint is more complicated, and dependent on the pMoA. Of course, for pMoAs which do not affect growth at all, no critical EMF can be found for any $x > 0$. For growth costs $\boldsymbol{S} = [0, 0, 1, s_R, s_H]$ the maximum growth effect of the toxicant is a complete cessation, and so the result is equivalent to (S23) with length L replacing cumulative reproduction. Under assimilation stress ($\boldsymbol{S} = [1, 0, s_G, s_R, s_H]$) $s_A \leq 1$ which limits shrinking to a maximum exponential decay rate $(r_B/y_P)t$. Similar arguments to those used in (S37) and (S48) gives

$$L(t_1;0) < \frac{100-x}{100} e^{\frac{r_B}{y_P}(t_E-t_1)} L(t_E;0). \tag{S24}$$

Finally, under maintenance stress ($\boldsymbol{S} = [s_A, 1, s_G, s_R, s_H]$) there is no maximum rate of shrinking. Therefore, as long as $t_1 < t_E$ any final size can be achieved. These results are summarised in Table 1. ☐

S2.4 Proof of Corollary 3.1

From Theorem 3 we know that $S(t;\alpha)$ and $R(t;\alpha)$ exist and are both continuous and strictly monotonically decreasing with respect to α. The integral of their product therefore also has these properties. It is also easy to check that 0% effects occur when $\alpha = 0$ and, since some α_1 exists which causes at least x% effects in one of the endpoints, then this α_1 must cause at least x% effects to B. ☐

S3 Non-uniqueness in a DEB model with reserve

The issues with elimination scaled by a surface area:volume (i.e. $\boldsymbol{X}_e = 1$) are not limited to DEBkiss models. Standard DEB-TKTD models also exhibit this when the feedback has the same structure. This is shown in Figure S1

for a stdDEB model.[7] However, in this case the model organism is unable to reallocate energy from the reproductive branch into the somatic branch, so shrinking is immediate once $dL/dt < 0$. The full equations for the physiological part of the model can be found in,[8] and the growth equation under starvation from the comments on Kooijman,[7] available from https://www.bio.vu.nl/thb/research/bib/Kooy2010_c.pdf.

S4 The pelagic microalgae model

The pelagic microalgae model of Weber et al.[9] was constructed to simulate the effects of variable exposures in a flow-through experimental setup designed to allow exposure profiles which match typical predictions made by environmental fate models. The model can be parametrized for multiple algal species.[9]

The exponential growth model is a system of ODEs to track the algal population density, A, in a volume with constant flow-through rate. The actual growth rate is reached by applying a series of inhibition functions to the maximum growth rate and subtracting algae lost through natural mortality and the amount lost at the end of the flow-through system. These inhibition functions all take the value one under optimal conditions, and drop towards zero as conditions worsen. Without toxic stress, algae finds an equilibrium population size which the nutrients in the system can sustain.

However, it has been noted that this flow-through experimental system is difficult to setup and maintain and thus it is difficult to obtain robust results.[10] Indeed, overcoming experimental challenges - not any modelling issue - was a major reason that EFSA determined that the algae population model was not ready for use in ERA.[10] Instead, a simplified version of the model which is directly applicable to the standard tests already used in lower tiers of ERA can be used. The main differences are that the algae are grown in static water and have a small initial biomass per unit volume of water.[11] As a result, algae are expected to grow exponentially without nutrient limitation becoming a factor. This means that the flow-through rate $D = 0$ and ignoring nutrient limitation through competition. This also removes two ODEs from the original model. It is probable that the full model of Weber et al.[9] provides a unique multiplier value. However, simulating nutrient limitation prevents a simple proof of this being achievable. Instead, we focus on the case where competition is ignored, but leave open the possibility for flowing water (i.e. $D \geq 0$). As this version of the model is also more amenable to use in ERA with EMFs, we feel that this does not weaken the result.

When there is no competition for nutrients the model equations simplify dramatically. There is only a single ODE

$$\frac{dA}{dt} = (\mu_{\max} f(C) g(T) h(I) - m_{\max} - D)A, \tag{S25}$$

where D is the flow rate of the water, $m_{\max}$ is the background mortality rate and $\mu_{\max}$ is the maximum growth rate. The functions, f, g and h are inhibition functions based on the external concentration of the toxicant, temperature and irradiation respectively. The temperature and irradiation inhibition functions are given in Weber et al.[9] As these functions are typically constant under laboratory conditions, and independent of the EMF α, we do not discuss them in detail.

At the Tier-2C level of ERA, model conditions (for temperature, nutrients and irradiation) will be held to the conditions experienced in the laboratory. Laboratory conditions can generally be considered ideal, or at least constant. In this paper we consider these inhibition functions as constant values. The exception to this is the inhibition caused by the toxicant concentration, described by the function

$$f(C) = \left(\frac{1}{1 + \left(\frac{C}{EC_{50}} \right)^b} \right), \tag{S26}$$

where C is the concentration affecting the algae and b is the slope of the dose response curve at the EC_{50}. Toxic stress is directly related to external concentration, i.e. the toxicokinetics are treated as instantaneous. It should be noted that (S26) differs from the equation often stated in the literature.[9,10] The chosen structure here better represents its use in the model. When $C = 0$ there is no inhibition to the growth rate (i.e. $f(0) = 1$). As $C \to \infty$ inhibition increases, causing $f(C)$ and the growth rate to decrease. Typically, the external concentration is used directly in (S26). However, for some substances a simple, one compartment scaled TK module is required. In these cases internal concentration is introduced as a second state variable, C_{int}, the generally used equation is

$$\frac{dC_{int}}{dt} = k_d(C - C_{int}), \tag{S27}$$

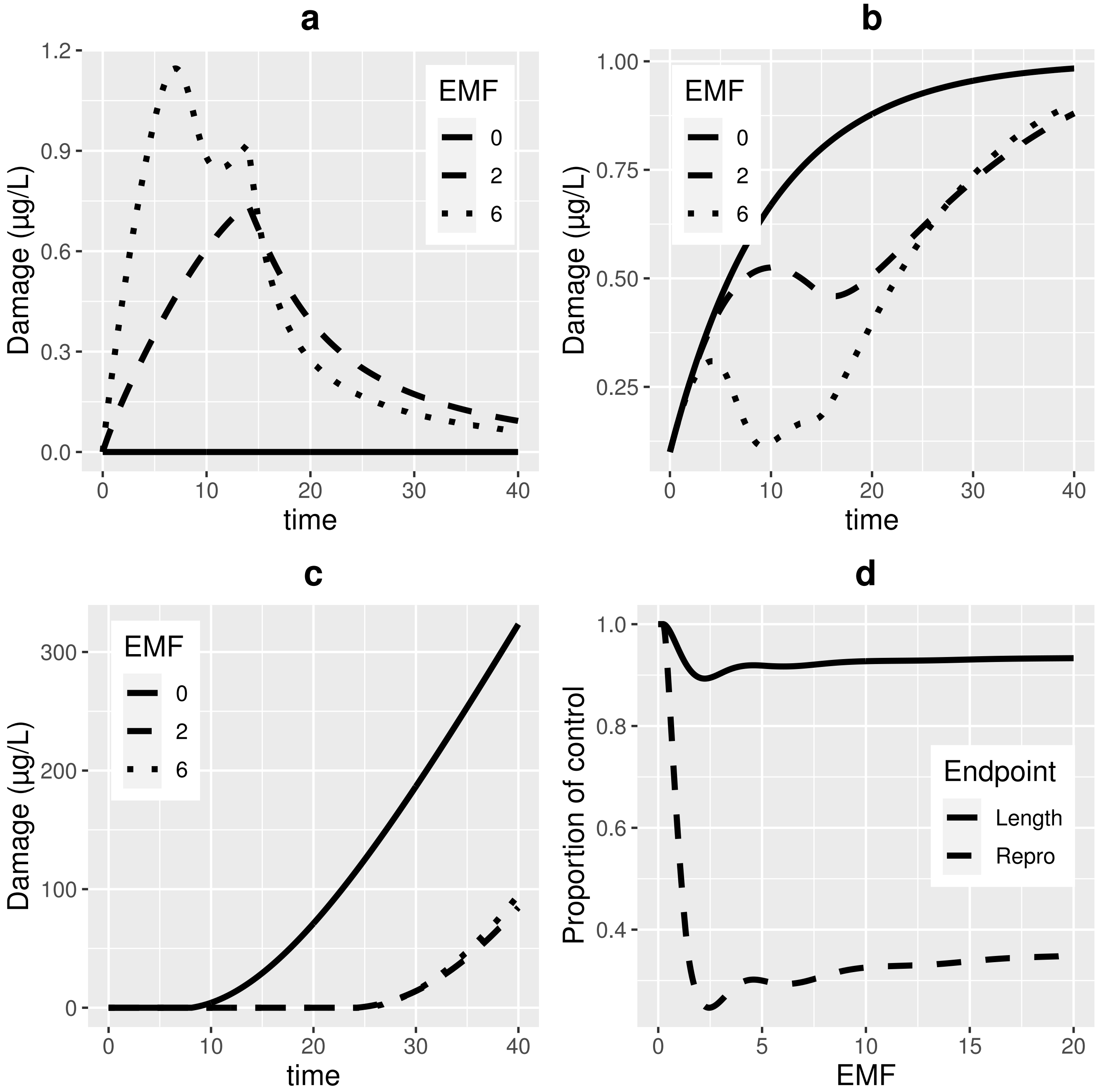

Figure S1: A recreation of Fig. 1 for a DEB model with a reserve. The (non-multiplied) exposure is a constant $1\mu g/L$ for the first 14 days and zero thereafter and effects assimilation only ($\boldsymbol{S} = [1, 0, 0, 0, 0]$). Parameter values match those in Fig. 1, with the additional parameters $\dot{v} = 0.6$, $\dot{k}_M = 0.6$ and $g = 1$. For the meaning of these parameters, see Jager et al.[8]

where k_d is the rate constant for the uptake and elimination of the compound. The internal concentration C_{int} replaces the external concentration C in (S26). Note that (S27) does not include dilution by growth, but that it could be introduced, similarly to the (scaled) damage ODE in DEB models.

Theorem S1. *Let $C_w(t)$ be a given non-zero exposure profile and $x > 0$ be the desired percentage effect (decreased algae) relative to control growth at some model end time t_E. $C_w(t)$ must be such that t_1, the first instance where the concentration is non-zero, satisfies*

$$A(t_1; 0) \leq \left(1 - \frac{x}{100}\right) A(t_E; 0) e^{(m_{max}+D)(t_E - t_1)}.$$

In addition, the external concentration cannot return to zero after t_1. Assuming optimum conditions throughout, we can pose this as a condition on either the latest that t_1 can be for a given x

$$t_1 = \ln\left(\left(\left(1 - \frac{x}{100}\right) e^{\mu_{max} t_E}\right)^{1/\mu_{max}}\right), \tag{S28}$$

or as an upper bound on the maximum x that can be reached for a given exposure profile

$$x = 100\left(1 - \frac{A(t_1; 0)}{A(t_E; 0)} e^{(m_{max}+D)(t_1 - t_E)}\right). \tag{S29}$$

Then, for some EMF α acting on $C_w(t)$, $A(t; \alpha)$ denotes the algal density at time t.

For any valid $x > 0$ percentage effect (exposure-induced mortality) there exists a unique EMF α_* such that

$$A(t_E; \alpha_*) = \left(1 - \frac{x}{100}\right) A(t_E; 0), \tag{S30}$$

this α_* is the EP_x for the exposure profile C_w.

The restriction introduced in (S28) arises because the only effect is to inhibit growth. As such, the maximum level of effect for any EMF is a complete stop to growth. Under such circumstances, biomass decays exponentially according to the sum of the mortality and flow-through rates. In order to reach x% effects by t_E some exposure must, at the latest, occur when a complete stop to growth will cause sufficient decay to reduce to (100-x)% of the control value at t_E. This is exactly what is described mathematically in (S28) and means that 100% effects cannot be guaranteed. In the model, this exponential decay never exactly reaches zero.

Unlike the upcoming Theorems, we also have the constraint that the concentration cannot return to exactly zero after t_1 in (S28). This is because this model has no internal concentration state variable, recovery of the growth rate is instantaneous with a reduction in concentration. On the other hand, with some damage or internal concentration state variable as seen in the DEB and *Lemna* models, or indeed in (S27), eliminating the substance takes time. Arbitrarily large EMF values induce greater damage which takes longer to eliminate and thus maintain the effect on the organism(s) for an arbitrary duration. It is therefore unimportant for those models whether or not the external concentration returns to zero.

S4.1 Proof of Theorem S1

Proof. The proof tracks the impact of the multiplier α through the model, showing that at each stage the model functions are continuous and monotonic with respect to α. Then, by the intermediate value theorem, any desired x% effect on algae (below the maximum possible level) corresponds to a unique multiplier value. Since, in the model, exposure can only inhibit growth, the maximal effect level is a complete cessation of growth from initial exposure to the end of the time window, with the associated decay through mortality and flow-through.

The external concentration $C(t)$ can either be directly specified or calculated via an ODE.[9] In the first case, continuity and monotonicity with respect to α follow immediately. For the second, as presented in Weber et al.[9] more work is required. The multiplier acts directly on the concentration entering the water body $C_w(t)$, which in turn acts upon the concentration in the water, $C(t; \alpha)$, through the ODE

$$\frac{dC(t; \alpha)}{dt} = \alpha C_w D - kC - DC \tag{S31}$$

Assuming the flow rate is constant, this ODE can be solved using the integrating factor method to give

$$C(t; \alpha) = \alpha D e^{-(k+D)t}\left[C_w(0) + \int_0^t e^{(k+D)\tau} C_w(\tau)\, d\tau\right] = \alpha C(t; 1) \tag{S32}$$

Where $C_w(0)$ is the initial concentration entering the system at time $t = 0$. Therefore C is clearly continuous and monotonically increasing with respect to α, since it has a linear effect on internal concentration. Note that $C(t;\alpha) = \alpha C(t;1)$ also holds when the external concentration is directly specified. Thus the remainder of the proof applies to both forms.

The inhibition function (S26) is now

$$f(\alpha C) = \left(\frac{1}{1 + \left(\frac{\alpha C}{EC_{50}} \right)^b} \right). \tag{S33}$$

This function is continuous with respect to $\alpha \geq 0$. To check the monotonicity, we differentiate (S33) with respect to α to reach

$$\frac{df(\alpha C)}{d\alpha} = -\frac{b\alpha^{b-1} \left(\frac{C}{EC_{50}} \right)^b}{\left(1 + \left(\frac{\alpha C}{EC_{50}} \right)^b \right)^2}. \tag{S34}$$

This is strictly negative for all positive values of α as long as $C > 0$. Therefore, increasing α will reduce (S33) and thus increase growth inhibition.

Finally, we must consider algal growth. Solving the ODE for growth gives

$$A(t;\alpha) = A(t_0;0) \exp \left(\int_{t_0}^{t} (\mu_{\max} g(T) h(I) f(Q) f(\alpha C) - m_{\max} - D) \, d\tau \right) \tag{S35}$$

The algae count shows exponential growth with a dynamic rate. Therefore it is a continuous function (in particular with respect to $f(\alpha C)$). Moreover, as α increases $f(\alpha C)$ will decrease, causing a decrease not only to the growth rate but also the overall algae at any point beyond the first time where the external concentration is non-zero.

The maximum effect level is to completely inhibit growth (i.e. $f(C) = 0$). When this occurs the algal population decays exponentially. As such, 100% effect on A relative to the control is impossible. However, for a given exposure profile we can calculate the maximum effect level. Assuming time t_1 as the earliest point where $f(C) = 0$ and $A(t_1;\alpha)$ as the algae level at that time the algal population for $t > t_1$ under continued zero growth is

$$A(t;\alpha) = A(t_1;\alpha) e^{-(m_{\max}+D)t}. \tag{S36}$$

In the most extreme case, $C = 0 \, \forall t < t_1$. For x% effects to be possible given this t_1 it must be the case that

$$A(t_1;0) \leq \left(1 - \frac{x}{100} \right) A(t_E;0) e^{(m_{max}+D)(t_E - t_1)}. \tag{S37}$$

Which can be rearranged to give t_1 or the maximum x as given in the Theorem.

As long as (S37) holds, α_* exists and is unique. □

S5 The *Lemna* model

The model of Schmitt et al.[12] is a mechanistic model describing the population growth of the duckweed *Lemna* sp. over time. The main state variable is dry weight biomass of *Lemna*, BM, and the main endpoint for ERA is a decrease in biomass relative to predicted control growth in the same environmental conditions. Similarly to the algae model it is a system of ODEs with inhibition functions describing the limitations imposed on growth by environmental conditions relative to the optimum. Biomass is lost according to the rate, k_{loss}, multiplied by the fraction of that maximum that is achievable, f_{loss}. These loss processes are the summation of mortality, respiration and other possible effects.

An inhibition function represents the reduced growth caused by the PPP. *Lemna* internal concentration over time, $C_{int}(t)$, is determined by a one compartment ODE describing uptake and elimination of the external concentration C_{ext}. The actual metric for inhibition caused by the PPP is the internal unbound concentration $C_{int_{unb}} := C_{int}/k_{p:w}$ where $k_{p:w}$ is a partition coefficient between plant and water. The inhibition function itself takes a similar form as that used in the algae model, but with a maximum effect level $E_{max} \leq 1$

$$f_{photo}^{C_{int_{unb}}}(t) = 1 - E_{max} \left(\frac{C_{int_{unb}}^b}{EC_{50_{int}}^b + C_{int_{unb}}^b} \right). \tag{S38}$$

The equations (S26) and (S38) are equivalent when $E_{max} = 1$.

The *Lemna* model of Schmitt et al.[12] is currently being reviewed and rewritten by an EFSA working group.[13] This refined description has already clarified and corrected the model. The full derivation and description of the model, and how it differs from the model of Schmitt et al.[12] is contained within their publication. The central state variables are the dry weight biomass BM and the internal mass of the toxicant M_{int} over time.

Environmental forcing variables such as temperature, irradiance and nutrient levels can all act as inhibitors on the growth rate through functions which take the value one under ideal conditions, and decrease towards zero as conditions worsen. The true inhibition level, $f_{photo}(t)$ is the minimum of each of these individual inhibiting functions. However, for our purposes we will assume ideal conditions, thus growth inhibition can only occur through stress caused by the internalised compound. The inhibition function in this case is:

$$f_{photo}(t) = f_{photo}^{C_{int_{unb}}}(t) = 1 - E_{max}\left(\frac{C^b_{int_{unb}}}{EC^b_{50_{int}} + C^b_{int_{unb}}}\right), \tag{S39}$$

where $C_{int_{unb}}$ is the unbound internal concentration. The unbound concentration is defined as $C_{int_{unb}} = C_{int}/k_{p:w}$ where $k_{p:w}$ is some partition coefficient. It is recommended that $k_{p:w} = 1$ is fixed in most cases. The model ODEs have the form

$$\begin{aligned}\frac{dM_{int}}{dt} &= P \cdot A(t)\left(C_{ext}(t) - \frac{C_{int}(t)}{k_{p:w}}\right) - M_{int}\frac{k_{met}}{k_{p:w}} - M_{int}k_{loss}f_{loss}(t)\\ \frac{dBM}{dt} &= \left(k_{photo\_max}f_{photo}(t) - k_{loss}f_{loss}(t)\right)BM(t).\end{aligned} \tag{S40}$$

Where P is the permeability, $A(t)$ is the surface area of the *Lemna* (which is related to the biomass by a constant ratio, $A = r_{A/dw}BM$), $C_{ext}(t)$ is the external concentration over time, k_{photo_max} is the maximum photosynthesis rate and k_{loss} is the sum of mortality, respiration and other processes which reduce biomass, for our purposes $f_{loss}(t)$ is a constant. Another useful quantity is the volume $V(t) = \frac{r_{fw/dw}}{d_{fw/V}}BM$, where $r_{fw/dw}$ and $d_{fw/V}$ are constant conversion factors.

Unsurprisingly, the similarity of the structures of the algae and *Lemna* models result in similarly structured theorems and proofs for the use of the mulplying factor method.

Theorem S2. *Assume constant ideal conditions for* Lemna *growth in a Laboratory setting. Let $C_{ext}(t)$ be a given non-zero exposure profile and $x > 0$ be some desired percentage effect (decreased* Lemna *biomass) relative to control growth at some model end time t_E. $C_{ext}(t)$ must be such that t_1, the first instance where the concentration is non-zero, comes before the point that* Lemna *biomass reaches*

$$\left(1 - \frac{x}{100}\right)BM(t_E;0)e^{f_{loss}(t_1)k_{loss}(t_E - t_1)},$$

which, assuming optimum conditions until first exposure, gives t_1 as

$$t_1 = \ln\left(\left(\left(1 - \frac{x}{100}\right)e^{(f_{photo}(t)k_{photo\_max})t_E}\right)^{1/(f_{photo}(t)k_{photo\_max})}\right) \tag{S41}$$

Alternatively, one can find the maximum x% for t_1 as

$$x = 100\left(1 - \frac{BM(t_1;0)}{BM(t_E;0)}e^{k_{loss}f_{loss}(t)(t_1 - t_E)}\right). \tag{S42}$$

Then, for some EMF α acting on $C_{ext}(t)$, $BM(t;\alpha)$ denotes the biomass at time t.

For any valid $x > 0$ percentage effect on biomass, and model end time t_E there exists a unique EMF α_* such that

$$BM(t_E;\alpha_*) = \left(1 - \frac{x}{100}\right)BM(t_E;0) \tag{S43}$$

this α_* is the EP_x value for the exposure profile C_{ext}.

S5.1 Proof of Theorem S2

For the purpose of the proof it is more convenient to deal with the dynamics of the internal concentration than the mass of the compound. We can derive these dynamics from (S40) using $C_{int} = M_{int}/V$ and that $\frac{dV}{dt} = \frac{r_{fw/dw}}{d_{fw/V}}\frac{dBM}{dt}$

$$\begin{aligned}\frac{dC_{int}}{dt} &= \frac{dM_{int}}{dt}\frac{1}{V} - \frac{r_{fw/dw}}{d_{fw/V}}\frac{M_{int}}{V^2}\frac{dBM}{dt} \\ &= \frac{P\cdot A(t)}{V}\left(C_{ext}(t) - \frac{C_{int}(t)}{k_{p:w}}\right) - \frac{M_{int}}{V}\frac{k_{met}}{k_{p:w}} - \frac{M_{int}}{V}k_{loss}f_{loss}(t) \\ &\quad - \frac{M_{int}}{V^2}\frac{r_{fw/dw}}{d_{fw/V}}\left(\left(k_{photo\_max}f_{photo}(t) - k_{loss}f_{loss}(t)\right)BM(t)\right) \\ &= \frac{P\cdot A(t)}{V}\left(C_{ext}(t) - \frac{C_{int}(t)}{k_{p:w}}\right) - C_{int}\frac{k_{met}}{k_{p:w}} - C_{int}k_{loss}f_{loss}(t) \\ &\quad - C_{int}\left(\left(k_{photo\_max}f_{photo}(t) - k_{loss}f_{loss}(t)\right)\right) \\ &= \frac{Pr_{A/dw}d_{fw/V}}{r_{fw/dw}}\left(C_{ext}(t) - \frac{C_{int}(t)}{k_{p:w}}\right) - C_{int}\frac{k_{met}}{k_{p:w}} - C_{int}k_{photo\_max}f_{photo}(t) \qquad \text{(S44)}\end{aligned}$$

Proof. We begin once again with the ODE for internal concentration (S44). Under some multiplier α the equation has the integral form

$$C_{int}(t;\alpha) = \alpha\frac{Pr_{A/dw}d_{fw/V}}{r_{fw/dw}}e^{(-\rho(t))}\left(C_{ext}(0;1) + \int_0^t e^{\rho(\tau)}C_{ext}(\tau)d\tau\right) = \alpha C_{int}(t;1), \qquad \text{(S45)}$$

where

$$\rho(t) = \frac{Pr_{A/dw}d_{fw/V}}{r_{fw/dw}k_{p:w}} + \frac{k_{met}}{k_{p:w}} + k_{photo\_max}f_{photo}(t).$$

As a product of exponential functions it is clear that (S45) is continuous with respect to α. The scaling transfers linearly from the external concentration to the internal concentration. Hence the internal concentration is monotonically increasing with respect to α, with strict monotonicity when $C_{int}(t;\alpha) \not\equiv 0$.

The internal concentration decreases the growth rate according to the inhibition equation $f_{photo}(\alpha C_{int_{unb}})$. This inhibition function is clearly continuous since $\alpha \geq 0$. Monotonicity can be seen through knowledge of the function's shape, but also by checking the derivative with respect to α

$$\frac{df_{photo}}{d\alpha} = -E_{max}\frac{b\alpha^{b-1}\left(\frac{C_{int_{unb}}}{EC_{50_{int}}}\right)^b}{\left(1 + \left(\frac{\alpha C_{int_{unb}}}{EC_{50_{int}}}\right)^b\right)^2}. \qquad \text{(S46)}$$

This is negative for all values of α, and zero when $C_{int_{unb}} = 0$. Hence monotonicity is shown. Finally, the ODE for biomass BM has the integral form

$$BM(t;\alpha) = BM(0;0)\exp\left(\int_0^t (k_{photo\_max}f_{photo}(\tau) - k_{loss}f_{loss}(\tau))d\tau\right) \qquad \text{(S47)}$$

As inhibition increases, f_{photo} decreases, so biomass at time t also decreases. Thus, for $\alpha_2 > \alpha_1$, $BM(t;\alpha_2) \leq BM(t;\alpha_1)$ with equality if and only if $C_{ext}(\tau) \equiv 0\,\forall\tau \in [0,t]$. Since we have specified $x > 0$ for the critical multiplier α_* there must be some exposure. Therefore, if α_* exists it must be unique.

As a final step to prove existence we must show that effects of at least $x\%$ are possible for some multiplier value. In the model, exposure can only affect the growth rate. When the growth rate is zero for some time t_1 onwards, $f_{photo}(t \geq t_1) = 0$ and from (S47) total biomass decays as

$$BM(t;\alpha) = BM(t_1;0)e^{-k_{loss}f_{loss}(t)(t-t_1)}.$$

Therefore, for $x\%$ effects to be possible relative to the control ($\alpha = 0$) some exposure must - at the latest - occur before total biomass exceeds

$$BM(t_1;0) = \left(1 - \frac{x}{100}\right)BM(t_E;0)e^{f_{loss}(t)k_{loss}(t_E-t_1)}, \qquad \text{(S48)}$$

assuming that f_{loss} remains constant throughout. Algebraic manipulation of (S48) reveals the expressions (S41) and (S42)). As long as (S48) holds, then a unique α_* exists for the given $x\%$ effect. □

Parameter	Meaning
	GUTS
k_u	substance uptake rate
k_e	substance elimination rate
k_r	damage repair rate
b_i	killing rate
z_i	threshold for toxic effects
h_b	background mortality
m_i	median of the distribution of thresholds
β	shape parameter for the distribution of thresholds
	DEB-TKTD
r_B	von Bertalanffy growth rate
f	scaled food availability
L_m	maximum structural length
L_p	structural length at puberty
h_b	background mortality
κ	allocation fraction to soma
y_P	yield for energy recovery in starvation
K_{RV}	Partition coefficient between egg and structure
F_{BV}	Egg dry weight relative to structure dry weight
k_d	dominant rate constant for scaled damage repair
b_b	effect strength of sublethal effects
b_s	killing rate
z_b	damage threshold for sublethal effects
z_s	damage threshold for mortality
s_i	stress factor for specific pMoA i
$\boldsymbol{S}$	binary vector showing all active pMoAs
x_i	feedback value for feedback i
$\boldsymbol{X_i}$	switch value for feedback process i, either active (1) or inactive (0)
$\boldsymbol{X}$	binary vector showing all active feedbacks
	Pelagic microalgae model
D	water flow rate
μ_{max}	maximum growth rate
m_{max}	background mortality rate
EC_{50}	concentration at which 50% effects occur
b	slope of the dose response curve at the EC_{50}
k_d	dominant rate constant
	Lemna model
E_{max}	maximum effect level
$\mathrm{EC}_{50_{int}}$	internal EC_{50}
b	slope of the dose response curve at the EC_{50}
P	permeability
k_{met}	metabolism rate
k_{photo_max}	maximum photosynthesis rate
k_{loss}	sum of all rates of processes which reduce biomass
$k_{p:w}$	partition coefficient between *Lemna* and water
$r_{fw/dw}$	fresh weight per dry weight
$d_{fw/V}$	fresh weight density

Table S1: Table of parameters for all models.

References

[1] Jager, T. & Ashauer, R. Modelling survival under chemical stress. A comprehensive guide to the GUTS framework. *Oakland, CA: Leanpub* (2018).

[2] Baudrot, V. & Charles, S. Recommendations to address uncertainties in environmental risk assessment using toxicokinetic-toxicodynamic models. *Scientific reports* **9**, 1–14 (2019).

[3] Jager, T. Revisiting simplified DEBtox models for analysing ecotoxicity data. *Ecological Modelling* **416**, 108904 (2020).

[4] Álvarez, O. A., Jager, T., Redondo, E. M. & Kammenga, J. E. Physiological modes of action of toxic chemicals in the nematode *Acrobeloides nanus*. *Environ. Toxicol. Chem.* **25**, 3230–3237 (2006).

[5] Ashauer, R. & Jager, T. Physiological modes of action across species and toxicants: the key to predictive ecotoxicology. *Environmental Science: Processes & Impacts* **20**, 48–57 (2018).

[6] Sherborne, N. & Galic, N. Modeling sublethal effects of chemicals: Application of a simplified dynamic energy budget model to standard ecotoxicity data. *Environmental Science & Technology* **54**, 7420–7429 (2020).

[7] Kooijman, S. A. L. M. *Dynamic energy budget theory for metabolic organisation* (Cambridge university press, 2010).

[8] Jager, T. & Zimmer, E. I. Simplified dynamic energy budget model for analysing ecotoxicity data. *Ecol. Model.* **225**, 74–81 (2012).

[9] Weber, D. *et al.* Combination of a higher-tier flow-through system and population modeling to assess the effects of time-variable exposure of isoproturon on the green algae *Desmodesmus subspicatus* and *Pseudokirchneriella subcapitata*. *Environmental toxicology and chemistry* **31**, 899–908 (2012).

[10] EFSA Panel on Plant Protection Products and their Residues (PPR) *et al.* Scientific opinion on the state of the art of Toxicokinetic/Toxicodynamic (TKTD) effect models for regulatory risk assessment of pesticides for aquatic organisms. *EFSA Journal* **16**, e05377 (2018).

[11] OECD. OECD guidelines for the testing of chemicals: Freshwater alga and cyanobacteria, growth inhibition test 201, adopted 23 march 2006, annex 5 corrected 28 july 2011. *OECD Publishing* (2011).

[12] Schmitt, W., Bruns, E., Dollinger, M. & Sowig, P. Mechanistic TK/TD-model simulating the effect of growth inhibitors on *Lemna* populations. *Ecological modelling* **255**, 1–10 (2013).

[13] Klein, J. *et al.* Refined description of the *Lemna* TKTD growth model based on Schmitt et al. (2013) – equation system and default parameters. *Report of the working group Lemna of the SETAC Europe Interest group Effect Modeling* (2021).
